# Transformer–CNN Hybrid Framework for Pavement Pothole Segmentation

**DOI:** 10.3390/s25216756

**Published:** 2025-11-04

**Authors:** Tianjie Zhang, Zhen Liu, Bingyan Cui, Xingyu Gu, Yang Lu

**Affiliations:** 1 Center for Advanced Infrastructure and Transportation, Rutgers University, Piscataway, NJ 08854, USA; tz367@soe.rutgers.edu (T.Z.); bingyan.cui@rutgers.edu (B.C.); 2 Department of Engineering, Boise State University, Boise, ID 83752, USA; 3 Institute of Space and Earth Information Science, Fok Ying Tung Remote Sensing Science Building, The Chinese University of Hong Kong, Hong Kong SAR, China; zhenliu@cuhk.edu.hk; 4 School of Transportation, Southeast University, Nanjing 211189, China; guxingyu1976@seu.edu.cn

**Keywords:** transformer, pothole, image segmentation, CNN, deep learning

## Abstract

**Highlights:**

**What are the main findings?**

- Developed a hybrid Transformer–CNN model (PoFormer) that significantly improves pavement pothole segmentation.
- Achieved higher detection accuracy than existing models under diverse environmental conditions.

**What is the implication of the main finding?**

- Enables more reliable and efficient pavement condition monitoring for intelligent transportation systems.
- Provides an open-source dataset to support further research and model development in road surface analysis.

**Abstract:**

Pavement surface defects such as potholes pose significant safety risks and accelerate infrastructure deterioration. Accurate and automated detection of such defects requires both advanced sensing technologies and robust deep learning models. In this study, we propose PoFormer, a Transformer–CNN hybrid framework designed for precise segmentation of pavement potholes from heterogeneous image datasets. The architecture leverages the global feature extraction ability of Transformers and the fine-grained localization capability of CNNs, achieving superior segmentation accuracy compared to state-of-the-art models. To construct a representative dataset, we combined open source images with high-resolution field data acquired using a multi-sensor pavement inspection vehicle equipped with a line-scan camera and infrared/laser-assisted lighting. This sensing system provides millimeter-level resolution and continuous 3D surface imaging under diverse environmental conditions, ensuring robust training inputs for deep learning. Experimental results demonstrate that PoFormer achieves a mean IoU of 77.23% and a mean pixel accuracy of 84.48%, outperforming existing CNN-based models. By integrating multi-sensor data acquisition with advanced hybrid neural networks, this work highlights the potential of 3D imaging and sensing technologies for intelligent pavement condition monitoring and automated infrastructure maintenance.

## 1. Introduction

Due to climate changes such as heavy snow and rainstorms, there are increasing pavement defects like potholes all over the world [1]. Potholes not only pose safety hazards to road users, but also contribute to accelerated pavement deterioration [2]. Accurately identifying and segmenting potholes from pavement images is crucial for effective road maintenance planning, targeted repairs, and resource allocation [3]. Pothole detection and maintenance are becoming of paramount importance in transportation infrastructure management [4]. There are a number of methods that can be used to inspect and detect potholes on road surfaces. These methods include visual inspection, automated inspection [5], ground-penetrating radar [6], light detection and ranging (LiDAR) [7], and infrared thermography [8,9]. By using these methods, pavement management agencies can identify and repair potholes before they become a hazard to drivers and cyclists. Traditionally, regular visual inspections are one of the most common methods for detecting potholes [10]. The pothole is visual inspected by professional engineers, but it is dangerous, labor-intensive, inefficient, and time-consuming [11]. Furthermore, visual inspection in pothole detection is subjective and very dependent on the experience and knowledge of the engineers [12]. Therefore, automated methods, including image processing techniques and machine learning-based methods, are emerged to solve this problem [13]. For example, Buza et al. [14] presented an unsupervised vision-based method by using image processing and spectral clustering to identify and estimate the location of potholes, but this method was only tested on 50 images and gained an accuracy of 81%. Koch et al. [15] proposed a novel method based on a histogram shape-based thresholding and morphological thinning and elliptic to automatically segment the potholes from pavement. However, methods based on conventional image processing and machine learning techniques often struggle to handle challenges due to complex variations in pothole shape, texture, and lighting conditions.

In recent years, deep learning models have emerged as a superior alternative, offering significant advantages over traditional image processing approaches [16,17,18]. Deep learning, powered by artificial neural networks with multiple layers, has revolutionized various fields, including transportation infrastructure detection [19,20,21]. The ability of deep learning models to automatically learn and extract meaningful features from data enables them to surpass the limitations of handcrafted features used in traditional methods [22]. Deep learning approaches, such as convolutional neural networks (CNNs), have demonstrated superior performance in a wide range of computer vision tasks, including object detection, defect classification, and semantic segmentation [23]. For example, Zhang et al. [24] proposed an ECSNet in segmenting the pavement distress in a real-time task. Ukawah et al. [25] used YOLOv3 SPP to detect the asphalt pavement pothole in a self-collected dataset, and the result showed a mean Pixel Accuracy (mPA) of 88.93%. Ahmed et al. [26] proposed a modified VGG-16 by removing some convolutional layers and changing different dilation rates as a backbone in a Faster RNN network. The results showed that the modified VGG-16 can achieve a higher Precision of 81.4% than VGG-16. The YOLOV5 with ResNet as its backbone achieved the highest mPA of 64.12%. These deep learning models leverage the hierarchical nature of neural networks to capture both low-level local features and high-level contextual information, enabling them to discern subtle patterns and distinguish between different types of distresses. However, there are disadvantages using pure CNN models. The most common architecture in deep learning models is the encoder–decoder structure [27], which CNN usually relies on. Given that CNNs have an architecture concentrating on localized features extraction, using a CNN as an encoder primarily focuses on local features and might miss out on the broader context, which is essential in differentiating potholes from other similar-looking features on a pavement [28]. Moreover, CNNs can easily overfit to specific textural patterns that occur in the training data, leading to less robust performance on unseen types of pavement or potholes with varying appearances. In addition to CNN models, several recent studies have explored emerging paradigms for pavement or material defect analysis. For example, biologically inspired approaches, such as spiking neural networks (SNNs), have been used for dynamic and energy-efficient feature extraction, demonstrating potential for real-time defect detection using time–frequency gradient analysis [29]. These methods highlight the growing diversity of intelligent defect analysis frameworks.

While deep learning models have yielded impressive results, recent advancements in the field of natural language processing have introduced a new paradigm: Transformers [30]. Transformers have revolutionized language modeling tasks by effectively capturing long-range dependencies and global context. This attention-based architecture has shown remarkable success in handling sequential data, and has recently been extended to image analysis tasks. For example, Dosovitskiy et al. [27] proposed a Vision Transformer model (ViT) on image classification tasks and it was compared to state-of-the-art convolutional networks, including ResNet and EfficientNet. The results showed that the proposed ViT performed better on datasets like ImageNet [31], CIFAR [32], and Oxford-IIIT Pets [33]. Zheng et al. [34] proposed a sequence-to-sequence structure, SEgementation TRansformer (SETR), to treat the image segmentation tasks. The Transformer layer was used to encode the images using a sequence of patches. This method reached a mean Intersection over Union (mIoU) of 82.15% in the Cityscape validation dataset [35]. These attempts showed that Transformer layers are good at encoding the information from the images at a high level of accuracy and efficiency without losing broader context details. Transformers have also started been utilized and applied in pavement crack detection. Han et al. [36] proposed Transformer FRTS to predict various asphalt pavement health indicators like rutting depth and surface texture depth. The inherent ability of Transformers to model and extract complex relationships and capture fine-grained details makes them well-suited for analyzing spatial patterns in pavement images. However, using Transformers as decoders can be dependent on the specific application and the architecture of the network. In some cases, they might not be as effective as other approaches, such as CNNs, particularly when it comes to local feature extraction and pixel-level prediction tasks [37]. For example, Guo et al. [38] presented a Crack Transformer by integrating a Swim Transformer as its encoder and a Multi-Layer Perception (MLP) as its decoder to detect and segment the pavement cracks rather than using a pure Transformer model. Crack datasets, including CFD [39] and Crack500 [40], were tested, showing that the Crack Transformer can better retrieve the defect length. Given the weaknesses of CNNs and Transformer, it is desirable to develop a new network model to form a stronger network that can avoid weakness and utilize the strength of both.

Thus, in this paper, we propose a novel approach, “PoFormer”, which is a Transformer–CNN hybrid model to leverage the power of Transformers and CNNs in pothole segmentation. In this model, the input images are sequenced first to several patches and then a Transformer-based encoder is adopted to extract the pattern and information from the patches. And then a decoder with four convolutional layers is utilized to take the encoded information and spatial features and reconstruct them into an output of the same size as the original image, but with segmented regions. By adapting this Transformer–CNN-based architecture specifically to pothole detection, we aim to overcome the limitations of traditional CNN-only methods, like limited contextual understanding and overfitting on textural features, to further improve the accuracy and robustness of pothole segmentation. Moreover, the Transformer’s ability to capture long-range dependencies and contextual relationships in images, and the convolutional layer’s ability to extract spatial hierarchies, are fully utilized in the model. Through extensive experimentation and comparisons with traditional deep learning models, we demonstrate the effectiveness of our proposed approach to accurately identify and delineating potholes from heterogeneous pavement surface images. Compared with existing hybrid Transformer–CNN segmentation architectures, such as UperFormer [37] and Crack Transformer [38], the proposed PoFormer introduces several distinct innovations. First, PoFormer adopts a Transformer-based encoder with 12 attention heads and multi-scale patch embedding to capture long-range contextual dependencies specific to heterogeneous pavement textures, which are often irregular and environment-sensitive. Second, unlike general-purpose hybrid models, PoFormer employs a lightweight CNN-based decoder with progressive upsampling and BatchNorm2D layers to recover fine-grained boundary details of potholes while minimizing over-segmentation. Third, we couple the network with a heterogeneous multi-sensor dataset, integrating high-resolution line-scan imagery and open source RGB images, which has not been explored in previous Transformer–CNN pavement studies. This combination of architectural adaptation and dataset diversity provides improved generalization for real-world pavement defect segmentation tasks.

In Section 2, we introduce the PoFormer architecture, the overall evaluation procedure, and the evaluation metrics we used in the work. Then, in Section 3, we present the statistics of the pothole dataset we collected and present the experimental results of testing models. Finally, we discuss the implications of our findings, and conclude with remarks on the potential and future directions of Transformer–CNN hybrid approaches for pavement analysis.

## 2. Methods

### 2.1. Pavement Distress Data Acquisition Vehicle

**Figure 1:**
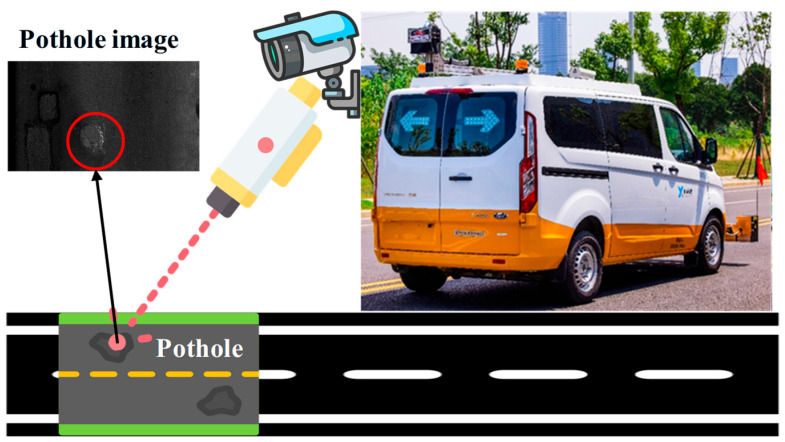
Pavement pothole inspection vehicle equipped with line-scan camera and infrared/laser lighting for high-resolution imaging.

To ensure the quality and diversity of the dataset, part of the images were newly collected by our team using a pavement distress inspection vehicle [41] as shown in Figure 1, while the rest were sourced from open access repositories. The inspection vehicle integrates a high-resolution line-scan camera and infrared/laser-assisted lighting, enabling continuous scanning of pavement surfaces under various environmental conditions. The system operates at normal driving speeds and provides millimeter-level resolution, capturing detailed surface defects such as potholes. These sensor-based data complement open source images, ensuring that the dataset reflects both controlled acquisition conditions and heterogeneous real-world scenarios.

### 2.2. PoFormer

**Figure 2:**
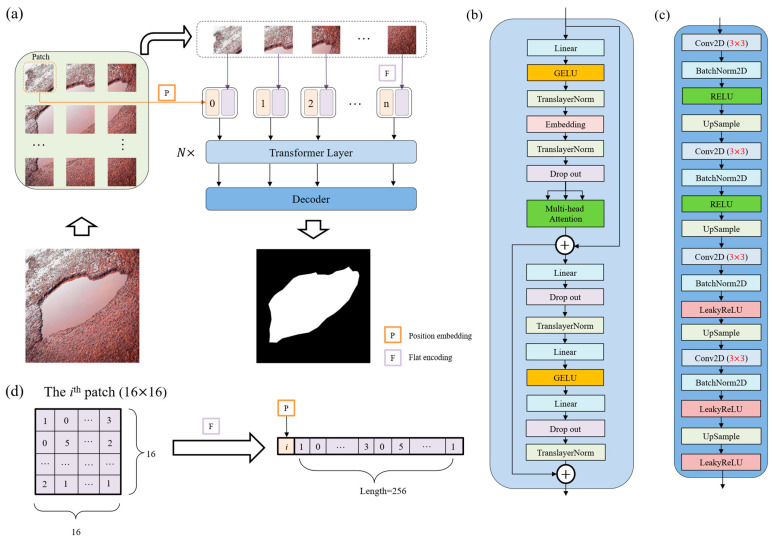
The structure of the proposed PoFormer and its components. (**a**) The overall workflow of the PoFormer model. (**b**) The Transformer Layer and utilizing it as the encoder in this model to extract the information from the images. (**c**) The structure of the Decoder, which is a CNN-based structure. (**d**) The flat encoding and position embedding process during image sequentialization; the number in the patch and sequence represents the pixel value.

Transformers and CNNs are two fundamental architectures in the field of deep learning. Transformers, known for their prowess in handling sequential data, primarily rely on a self-attention mechanism that allows for them to weigh the importance of different parts of the input data. They can capture and utilize relationships between elements in a sequence that are separated by considerable distances. Given the complex pavement service status, pothole detection is not limited to recognizing the pothole itself, but rather to understanding its context within the surrounding pavement, which may include varying textures and conditions. This holistic view allows for more accurate and robust segmentation. CNNs are well-established in image processing for their ability to extract the spatial hierarchies of features due to their convolutional layers, which are crucial for accurately identifying and delineating the shapes and boundaries of potholes in varied pavement conditions. Combining a Transformer as the encoder and a CNN as the decoder in one model, we aim to use the advantages of both. Thus, a Transformer–CNN hybrid model, PoFormer, is proposed in this work to segment the pothole from the pavement. The proposed PoFormer model is designed to enhance the performance of pavement pothole detection through enlarging the receptive fields of the CNN-based part and compensating the Transformer part for the loss of local fine-grained contextual information. The neural network architecture of the proposed PoFormer model is shown in Figure 2.

As illustrated in Figure 2a, the proposed PoFormer architecture consists of an image sequentialization part, an encoder, and a decoder. The resolution of images in this dataset ranges from 200×133 pixels to 4032×3024 pixels, as the images are collected from different resources. Hence, the input images would be resized to 256×256 first before performing the image sequentialization, as the model requires a fixed-size input, which is crucial for batch processing. Moreover, resizing to 256×256 helps to reduce the computational burden, making the training and inference processes more efficient. Because the Transformer layer asks for sequence data as its input, the resized 2D images would be transferred to one-dimensional sequences to meet the requirement of the input to a Transformer layer. The model intakes a pavement image and partitions it into *n* patches (in this case, each patch has a resolution of 16×16; therefore, *n* equals 256). As shown in Figure 2d, these patches undergo a linear transformation to obtain flat encodings (*F*) with an added positional embedding (*P*), formalized as *P*(*i*) + *F*(*p_i_*), where *p_i_* is the *i*th patch and *P*(*i*) is its corresponding positional embedding. This positional information is vital in maintaining the geometric fidelity of the patches, which is paramount in accurately segmenting the intricate features of potholes.

In the encoder, as shown in Figure 2b, the patch embeddings are processed through *N* Transformer layers (*N* = 12 in this case). Each layer is composed of a multi-head attention (*A*) and a position-wise feed forward network (*F*), where residual connections and layer normalization (TranslayerNorm) are applied before each sub-layer. Multi-head attention [27] is utilized in the encoder to capture complex dependencies and handle sequential data. Note that the multi-head attention is an extension of the attention mechanisms. It allows for the Transformer to jointly attend to information from different representation sub-spaces at different positions. The multi-head attention can be defined as Equation (1):

(1) $$A=Concat({head}_{1}\ldots ,{head}_{h})W^{o}$$

(2) $${head}_{i}=Attention\left({QW}_{i}^{Q},{KW}_{i}^{K},{VW}_{i}^{V}\right)$$

where each ${head}_{i}$ is computed, enabling the model to discern diverse feature interdependencies within the pavement image (h is defined as 12 in this model). $W^{o}$ stands for the output weight matrix to produce the final output, which carries the contextually enriched information. ***Q***, ***K***, and ***V*** stand for Query, Key, and Value, respectively. Query represents the set of vectors that are used to query the data. Keys are paired with values and are used to retrieve them. The similarity of a query with the keys determines how much attention is given to the corresponding values. Values are the actual content that you want to retrieve. The matrices $W_{i}^{Q}$, $W_{i}^{K}$, and $W_{i}^{V}$ are learned weight matrices that transform the input representations into the respective queries, keys, and values for the *i*th attention head. Each head in multi-head attention has its own set of these weight matrices, allowing for the model to attend to different parts of the input in different ways simultaneously. $Attention\left({QW}_{i}^{Q},{KW}_{i}^{K},{VW}_{i}^{V}\right)$ can be calculated using Equation (3):

(3) $$Attention\left(Q,K,V\right)=softmax(\frac{QK^{T}}{\sqrt{d_{k}}})V$$

where $d_{k}$ is the scaling factor, which is the dimensionality of the keys and queries, used to avoid overly large dot products. *T* means the transpose operation. The SoftMax function is applied to the result of the scaled dot-product, providing a probability distribution.

Queries, keys, and values in the attention mechanism are vectors that represent different aspects of each patch in the input image. In the context of pothole segmentation, queries could be understood as representations of what the model is looking for (e.g., edges, textures associated with potholes), keys as aspects of the current patch that could be relevant to the search, and values as the actual content of the patch. In our proposed PoFormer model, the attention mechanism allows for the model to focus on specific parts of the pavement images that are more relevant for identifying potholes, giving it the capability to distinguish between potholes and other similar-looking features on the road by learning the most pertinent features to pay attention to for the task at hand.

Within the Transformer layers, the feed-forward network employs Gaussian Error Linear Units (GELU) [42] as the activation function, facilitating the model’s ability to represent complex mappings. The network is formalized in Equation (4):

(4) $$F(x)=GELU(xW_{1}+b_{1})W_{2}+b_{2}$$

which introduces necessary non-linearity into the feature transformation process. $W_{1}$, $W_{2}$ are the weights of layers; $b_{1}$ and $b_{2}$ are the bias of layers. GELU is utilized as the non-linear activation function in the encoder part. The equation of GELU is shown in Equation (5):

(5) $$GELU\left(x\right)=x\phi (x)$$

where ϕ(x) is the cumulative distribution function for gaussian distribution.

The feed-forward network within the Transformer layers further processes these features, employing the GELU activation function to introduce non-linearity, enhancing the model’s ability to represent complex functions. The inclusion of dropout and layer normalization at each step mitigates the risk of overfitting and ensures stable learning, both of which are vital for the model to generalize well across varied pavement conditions.

The decoder architecture is illustrated in Figure 2c. During training, the distribution of inputs to each layer changes due to adjustments in the parameters of the preceding layer. This slows down the training process because each layer requires re-initialization of its parameters. To address this, we apply 2D batch normalization (BatchNorm2D) [43] after each convolutional layer in the decoder. The batch normalization operation is defined in Equation (6):

(6) $$y=\frac{x-\mu }{\sqrt{\sigma^{2}+\epsilon }}\times \gamma +\beta$$

where *x* is the input, y is the output, γ and β are learnable parameters, μ and $\sigma^{2}$ are the mean and variance of *x*, and ϵ is a small constant added for numerical stability (set to $e^{-5}$ here).

The decoder utilizes a succession of upsampling and convolutional operations to translate the high-level Transformer features into a segmented image. The upsampled feature map is convolved with learned filters $W_{conv}$ followed by batch normalization and activation functions, encapsulated as $\phi (B(Conv(U*W_{conv}+b_{conv})))$. This sequence is iterated to refine the segmentation granularity progressively, as shown in Equation (7):

(7) $$s=\;\phi (B(Conv(U*W_{conv}+b_{conv})))$$

where *s* represents the sequence in decoder, U stands for the upsampled feature map, *B* represents the Batch Normalization, *ϕ* is the activation function, *Conv* stands for the convolutional layer, and $W_{conv}$ and $b_{conv}$ are the weights and biases of each convolutional layer. ∗ represents 2D convolution.

RELU and LeakyRELU are used as the activation function in the decoder part. The equations are shown below.

(8) $$ReLU\left(x\right)=\left\{\begin{array}{c} x,\;\;x\geq 0\\ \;\;\;\;\;\;0,\;\;otherwise \end{array}\right.$$

(9) $$LeakyReLU\left(x\right)=\left\{\begin{array}{c} x,\;\;x\geq 0\\ \;\;\;\;\;ax,\;\;otherwise \end{array}\right.$$

where *a* controls the angle of the negative slope and $a=e^{-2}$ in this case. The LeakyReLU activation function can improve model fitting with minimal added computational cost and risk of overfitting.

Incorporating these academic notations and formulations equipped the model description with the necessary rigor for pavement surface pothole inspection.

### 2.3. Overall Evaluation Procedure

In order to evaluate the performance of the proposed method, five well-established models were evaluated alongside our novel Transformer–CNN hybrid model. The models under scrutiny are as follows: U-Net [44], LRASPP [45], FCN [46], E-Net [47], and AttuNet [48]. These models were selected based on their prominence in the field and their varied architectural principles, which include the following:

(1) U-Net: A convolutional network renowned for its efficacy in biomedical image segmentation.

(2) LRASPP: An adaptation of the Lite Reduced Atrous Spatial Pyramid Pooling (R-ASPP) Network, employing a MobileNetV3-Large backbone, optimized for mobile devices.

(3) FCN: A fully convolutional network, herein utilizing a ResNet-50 [49] backbone, known for its deep residual learning framework.

(4) E-Net: A network architecture designed for real-time semantic segmentation, as originally proposed by Paszke et al. [47], using the default hyperparameter settings.

(5) AttuNet: Specifically designed for scenarios with limited training samples, this model emphasizes attention mechanisms to refine feature representations.

Although these models were originally proposed several years ago, they remain the dominant benchmarks for pixel-level pavement distress segmentation tasks due to their balance of computational efficiency and accuracy. Recent studies [38,48] have continued to employ U-Net, FCN, LRASPP, E-Net, and AttuNet as reference baselines to ensure consistency and comparability in evaluating new algorithms. These architectures represent different design philosophies: FCN and U-Net as early encoder–decoder models, E-Net for real-time segmentation, LRASPP for mobile efficiency, and AttuNet for attention-based enhancement under limited data. Therefore, including these models provides a comprehensive and standardized baseline for assessing the performance of the proposed PoFormer.

**Figure 3:**
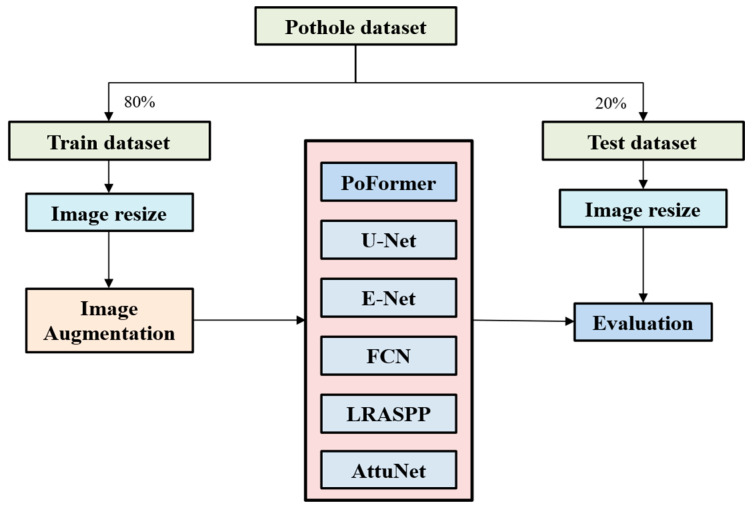
The overall procedure of model performance evaluation.

A self-collected dataset is proposed to evaluate the performance of these models. The dataset has been published on GitHub Repository (https://github.com/tjboise/PoFormer, accessed on 2 November 2025). In this dataset, a total of 1183 images featuring road surface potholes have been compiled, some sourced from the internet (920 images) and others collected by our team (263 images). Thus, it contains heterogeneous features which can evaluate the generalization of the model in complex situations. To ensure statistical robustness, the original dataset underwent a randomized tripartite split, forming the basis for three independent training and testing cycles. This methodology enabled the computation of average performance metrics and associated standard deviations, which can show the robustness of the model’s performance. The dataset was partitioned into training and testing subsets in an 80:20 ratio. Given the heterogeneous image resolutions stemming from varied acquisition sources, a standardization procedure resized all images to 256 × 256 pixels prior to model training. Data augmentation techniques, including random resizing and horizontal flipping [50], were systematically applied to the training images and their corresponding ground truths. Such augmentation generated novel inputs for the neural networks, enhancing the model’s performance by leveraging the increased quantity and diversity of the dataset. Subsequent to augmentation, the training images were converted to grayscale to streamline the learning process. The step-wise of the model performance evaluation procedure is shown in Figure 3.

Root Mean Squared Propagation (RMSProp) [51] was employed as the optimizer across all models to facilitate parameter updates. The ways to update the weights in the models are shown in Equations (10) and (11):

(10) $$w\left(t+1\right)=w\left(t\right)-\frac{\eta }{\sqrt{v\left(t\right)+\epsilon }}g\left(t\right)$$

(11) $$v\left(t\right)=\beta \cdot v\left(t-1\right)+(1-\beta )\cdot {\left(g\left(t\right)\right)}^{2}\;$$

where ***w***(*t*) is the model parameter at *t*th iteration, ***v***(t) is the squared gradient moving average at time step, *g*(t) is the gradient, and *η* is the learning rate. *β* is the decay rate (*β* = 0.9). ϵ is a small scalar (e.g., 10^−8^) used to prevent division by zero.

RMSProp is an adaptive learning rate optimization algorithm which was designed to address the diminishing learning rate issue of the Adaptive Gradient Algorithm. In RMSProp, the learning rate is adjusted on a per parameter basis, depending on the importance of the parameters. The training regimen consisted of 300 iterations for each network, with an initial learning rate set at 1 × 10^−3^ and a batch size of 16. Classification thresholds were standardized; an output value exceeding 0.5 denoted a pothole, while values below indicated background. The loss function utilized was BCEWithLogitsLoss [52], as shown in Equation (12), which amalgamates a sigmoid activation with Binary Cross Entropy loss, offering enhanced numerical stability over the conventional approach of a standalone Sigmoid activation followed by BCELoss [48].

(12) $$L_{BCE}=-\frac{1}{N}\sum_{i=1}^{N}\left[y_{i}log\sigma \left(z_{i}\right)+(1-y_{i})log(1-\sigma \left(z_{i}\right))\right]$$

where $\sigma \left(z_{i}\right)$ is the sigmoid function.

To ensure a fair comparison, all baseline models (U-Net, FCN, LRASPP, E-Net, and AttuNet) and the proposed PoFormer were trained and evaluated under identical experimental configurations. Each model used the same dataset split (80% for training and 20% for testing), input resolution of 512 × 512 pixels, and BCEWithLogitsLoss as the objective function. The RMSProp optimizer with an initial learning rate of 1 × 10^−4^ and batch size of 8 was applied consistently across all models. The learning rate decayed by a factor of 0.98 every 10 epochs, and training was conducted for 100 epochs. All implementation details followed either the original architecture papers or their official PyTorch implementations to ensure consistency and reproducibility.

In this work, all experiments were implemented in Python 3.10 using the PyTorch 2.2.0 framework with CUDA 12.1 and cuDNN 8.9 support. Model training and testing were performed on a workstation equipped with an Intel(R) Core(TM) i9-10900X CPU (Intel, Santa Clara, CA, USA), an NVIDIA RTX A4000 GPU (16 GB memory, Nvidia, Santa Clara, CA, USA), and 64 GB RAM, running Windows 10 Professional (64-bit).

### 2.4. Evaluation Metrics

We evaluate the performance of each model using Precision (P), Recall (R), F_1_ score, Mean Intersection over Union (mIoU), and Mean Pixel Accuracy (mPA). The F_1_ score is calculated as the harmonic mean of Precision (P) and Recall (R), according to Equation (13):

(13) $$F_{1}=\frac{2PR}{P+R}$$

Precision and Recall are defined in Equations (14) and (15).

(14) $$P=\frac{TP}{TP+FP}$$

(15) $$R=\frac{TP}{TP+FN}$$

where TP, FP, and FN represent the number of true positives, false positives, and false negatives, respectively.

To assess the performance of the segmentation models, we employed mIoU and mPA as the principal metrics. The IoU metric quantifies the percentage overlap between the model’s predicted pothole segmentation and the ground truth. mIoU is less biased towards the size of the pothole. It treats large and small potholes more equally, which is important in a real-world scenario where potholes vary significantly in size. It is calculated for each class separately within segmentation tasks according to the following equation:

(16) $$IoU=\frac{A_{o}}{A_{u}}$$

where $A_{o}$ denotes the area of overlap between the predicted segmentation and the ground truth, and $A_{u}$ stands for the combined area of the predicted and ground truth segmentations.

The mIoU represents the mean of IoU scores across all classes. It is determined by averaging the IoU for each class, as expressed in Equation (17):

(17) $$mIoU=\frac{1}{n}\sum_{i=1}^{n}{IoU}_{i}$$

Here, *n* is the number of classes within the dataset, which in our case is two (pothole and non-pothole areas).

Pixel Accuracy (PA) is another straightforward metric, defined as the ratio of correctly classified pixels to the total pixel count. For an individual class, PA is given by

(18) $$PA=\frac{P}{T}$$

where *T* is the total pixel count that should be classified within that class and *P* is the number of pixels accurately classified as belonging to that class.

mPA is the average of the PA values across all classes, calculated using the following formula:

(19) $$mPA=\;\frac{1}{n}\sum_{i}^{n}{PA}_{i}$$

While mIoU focuses on the overlap of the segmented area, mPA gives insight into the overall accuracy across all pixels. The utilization of mIoU and mPA offers a balanced evaluation of the models, considering both the Precision of the segmentation and the Accuracy of the pixel classification, thereby providing a comprehensive view of the model’s segmentation performance. While the PA and mPA indices are standard image segmentation metrics, their engineering relevance lies in quantifying the reliability of pavement surface condition assessment. In road maintenance practice, accurate pixel-level classification enables precise estimation of the defect area, proportion, and severity, which are critical indicators for maintenance prioritization and resource allocation. Therefore, PA serves as an indirect measure of how accurately the system can assess the extent of pavement deterioration, supporting data-driven decision-making in pavement management systems.

## 3. Results

### 3.1. Dataset Characteristics for Open Source Data Access

**Figure 4:**
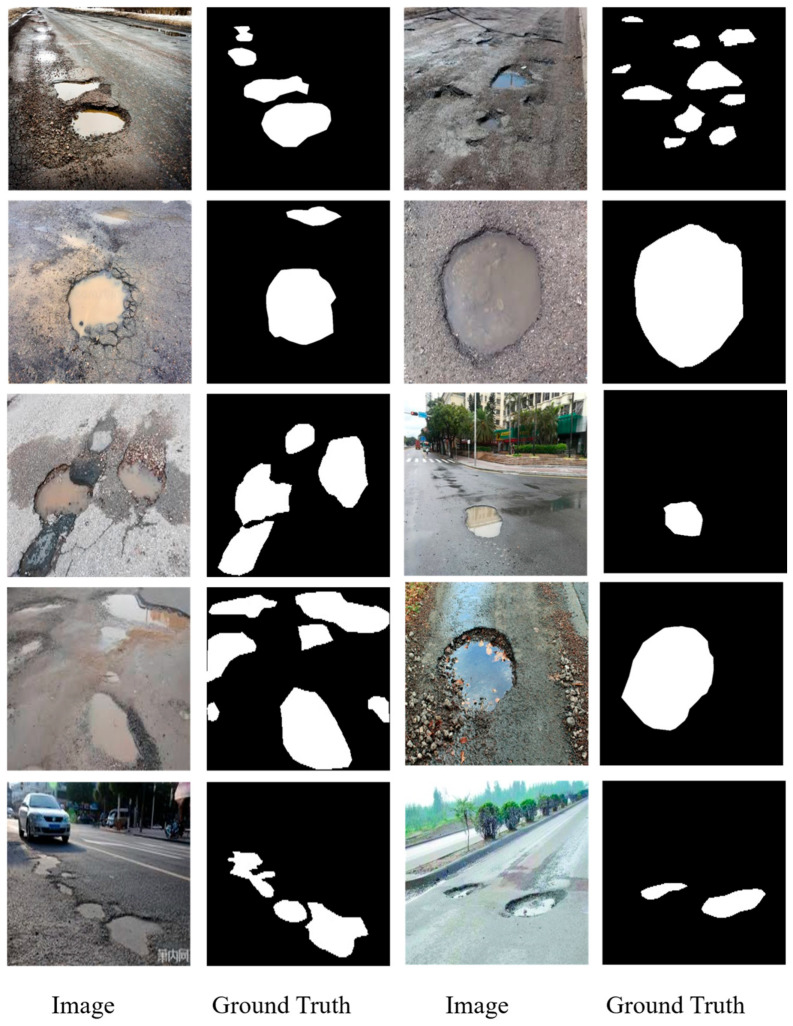
Some representative samples in the proposed dataset.

Prior studies have often developed bespoke methods tailored to small, proprietary datasets to validate their accuracy. This practice, however, significantly hampers the comparability of various approaches. Consequently, we have established a comprehensive open source database to serve as an empirical foundation for advancing research in pothole detection and segmentation. The dataset comprises 1183 manually annotated RGB images of potholes, ensuring a high level of detail and accuracy in the annotations. The final dataset used in this study combines 263 pothole images from the open source PaveDistress dataset and 920 images collected from publicly available internet sources. Unlike the PaveDistress dataset, which primarily features isolated pavement views, the internet-sourced images include diverse and complex backgrounds such as vehicles, pedestrians, vegetation, and roadside buildings. Some representative images and their corresponding segmentations are shown in Figure 4.

Figure 4 illustrates a curated selection of images from our comprehensive dataset, which is designed to reflect a range of real-world conditions affecting pothole visibility and detection. For example, the pothole easily collects water. The road condition could be complex; vehicles, people, trees, and buildings all appear in the background. This is also the reason why we collected heterogeneous features in the dataset. Accompanying each image is its respective binary segmentation mask, delineating the pothole boundaries for computer vision applications. This collection showcases various pothole scenarios, including different sizes, shapes, and severities, as well as a variety of environmental conditions such as wet and dry pavements. Most of the images contain more than one pothole, and each one can be a random size and irregular shape with wet or dry conditions, which demonstrates the strong heterogeneity features of pothole morphology distribution. The images originate from a dual-sourced methodology: the first set is derived from an extensive internet search to ensure diversity, and the second set consists of photographs taken manually by the research team, aiming to capture the nuanced details of potholes in local environments. This integrative approach can increase the difficulties when the model learns the features of the different images, such as different resolutions and varying pixels contained in the pothole images. However, this kind of heterogeneous dataset can ensure the its robustness and utility in developing and benchmarking pothole detection algorithms. Researchers and practitioners can access the full dataset, which contains heterogeneous images and precise annotations, on the GitHub repository at https://github.com/tjboise/PoFormer (accessed on 2 November 2025). The availability of this dataset aims to foster innovation and standardize benchmarks in the domain of automated pothole detection and segmentation, being our intended contribution to the field of pavement defect segmentation and the data science community.

**Figure 5:**
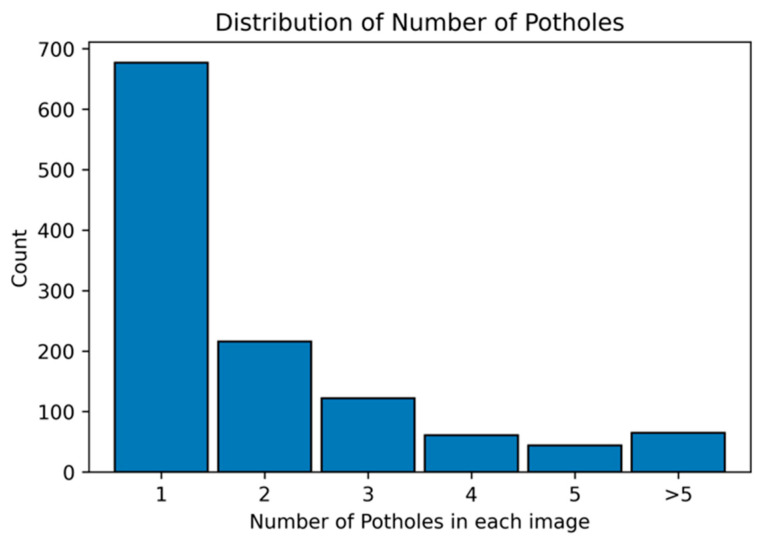
Distribution of pothole frequencies per image in the dataset.

Figure 5 provides a quantitative analysis of the pothole frequencies observed in the dataset. It delineates the count of potholes per image, offering insights into the prevalence and clustering of potholes within individual images. Specifically, the dataset comprises 677 images, each depicting a single pothole, highlighting the most common occurrence in urban settings. In contrast, a significant number of images, amounting to 506, feature more than two potholes. A smaller subset of 65 images captures scenarios with over five potholes, illustrating more severe roadway degradation. This variation in pothole distribution is reflective of actual road conditions and is critical for ensuring the diversity needed for training computer vision models. The wide range of pothole manifestations within the dataset is intended to bolster the generalization capabilities and enhance the robustness of pothole detection models when applied to real-world scenarios.

**Figure 6:**
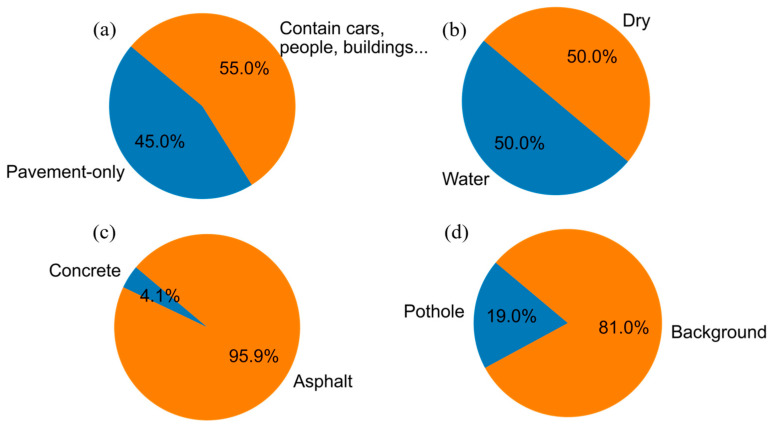
Compositional statistics of the pothole detection dataset. (**a**) pavement-only vs. complex scenes; (**b**) wet vs. dry pavement; (**c**) concrete vs. asphalt surfaces; (**d**) pothole vs. non-pothole pixel ratio.

Figure 6 provides a comprehensive breakdown of the dataset’s composition in terms of the various environmental and contextual factors relevant to pothole detection. Figure 6a contrasts the proportion of images featuring solely pavement backgrounds (45%) with those depicting additional elements such as vehicles, pedestrians, buildings, and vegetation (55%), underscoring the complexity the model must navigate. The presence of water in potholes, a factor influencing the performance of detection algorithms, is accounted for in Figure 6b, which equally splits the dataset between wet (50%) and dry (50%) pavement conditions, thereby enhancing the robustness of the resulting model. Figure 6c differentiates between concrete (4.1%) and asphalt (95.9%) pavements, with the latter being more predominant, mirroring the common road compositions encountered in urban environments. Lastly, Figure 6d quantifies the pothole to non-pothole pixel ratio within the images, revealing that potholes account for an average of 19% of the image area, a statistic that further informs the severity of road damage represented in the dataset.

### 3.2. Model Performance

**Figure 7:**
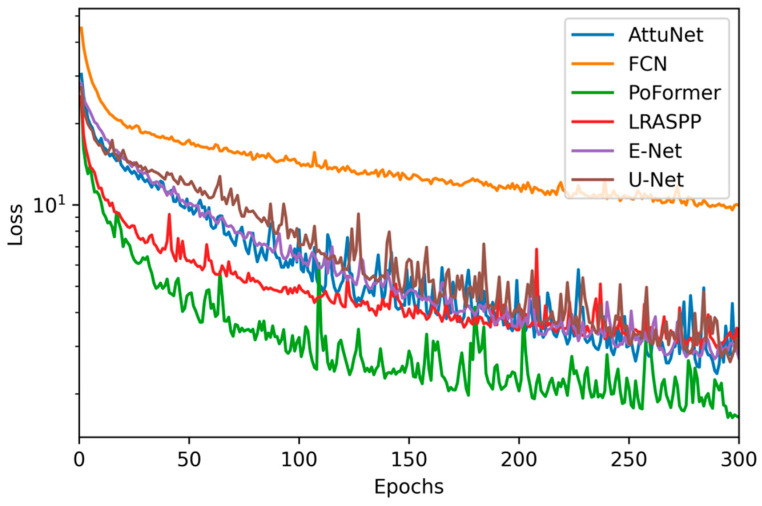
Epoch-wise loss trajectories for evaluated models.

Figure 7 presents the epoch-wise progression of the loss function for each evaluated model throughout the training phase. It is observed that PoFormer consistently maintains the lowest loss, indicative of superior model convergence and learning efficacy. In contrast, the FCN model exhibits the highest loss values across epochs, which correlates with its suboptimal performance on the test data. This inverse relationship between training loss and test performance suggests that PoFormer is better suited for capturing the nuances of the task, whereas the FCN model’s learning trajectory indicates potential challenges around generalization or model capacity.

Table 1 delineates a detailed comparative assessment of the evaluated models, including the proposed PoFormer, across key performance metrics including Precision, Recall, and F_1_ score.

The data reveals that, while the U-Net model achieves the pinnacle of Precision, it falls short in terms of Recall, culminating in a diminished F_1_ score. Contrariwise, the PoFormer model excels, registering the most substantial Recall and F_1_ scores among its counterparts. This suggests that PoFormer balances the trade-off between Precision and Recall more effectively, potentially rendering it a more robust solution for the given application.

**Figure 8:**
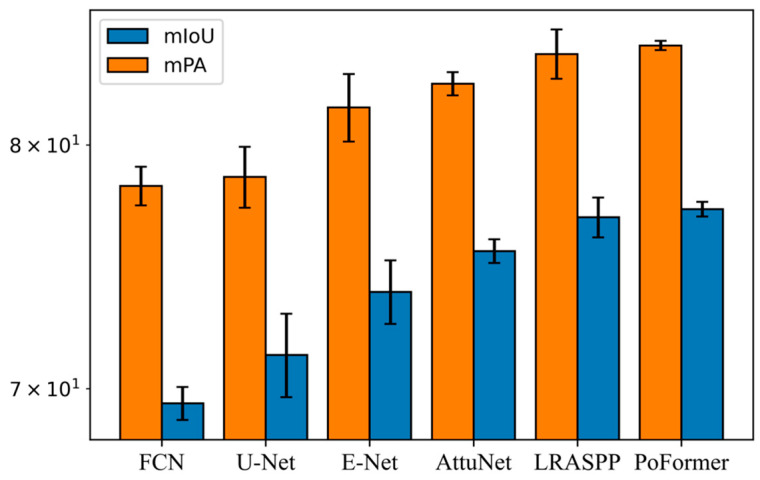
Comparative analysis of mIoU and mPA metrics across models.

Figure 8 presents the performance of various models evaluated in terms of mIoU and mPA. Each model was evaluated three times on distinct subsets randomly sampled from the full dataset. The mean and standard deviation of the performance metrics were then calculated across these three evaluation runs.

The FCN registered an average mIoU of 69.44 (±0.63) and an average mPA of 78.23 (±0.83), reflecting the most consistent but worst performance among all the models. The LRASPP model achieved the second place in both mIoU and mPA, achieving an mIoU of 76.89 and an mPA of 84.09. It performs slightly less well than the proposed PoFormer. Our proposed method performed the highest accuracy among all of the evaluated models, with an mIoU of 77.23 (±0.31) and an mPA of 84.48 (±0.22). The standard deviation is lower than other models. This means that PoFormer’s performance is indicative of its both robustness and precision in segmenting potholes within the dataset.

**Figure 9:**
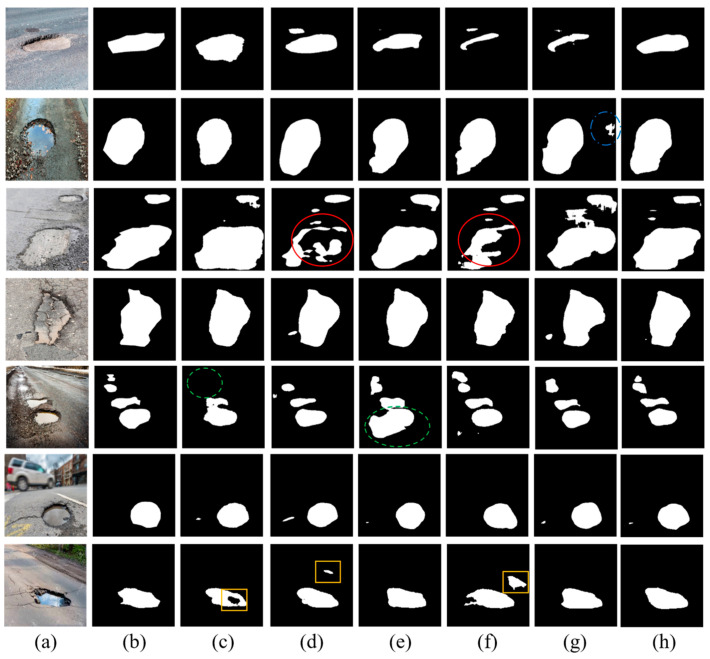
The visualization of detection results of compared methods: (**a**) original image; (**b**) ground truth; (**c**) E-Net; (**d**) FCN; (**e**) LRASPP; (**f**) U-Net; (**g**) AttuNet; (**h**) PoFormer.

To compare the final segmentation performance, Figure 9 visualizes example detection results for each of the deep learning-based models.

As we can see from Figure 9, it is discernible that the FCN and U-Net models exhibit discontinuities in segmentation, particularly highlighted within the regions enclosed by red circles. These areas demonstrate a lack of alignment with the ground truth, with the models producing fragmented segmentations. The green-circled instances in the fifth row bring to light the limitations of E-Net, which omits smaller potholes. Although the F_1_ score of LRASPP is close to the PoFormer, the Recall value is 71.91, which is not as high as the prediction results from PoFormer (72.20). This means the LRASPP tends to erroneously classify background pixels as potholes, as we can see from the green dashed circle in the Figure 9e. The predicted pothole area in LRASPP is 58.7% larger than the ground truth, while for the PoFormer, this value is approximately 6.9%. The segments marked by yellow rectangles accentuate the propensity of both FCN and U-Net to engage in over-segmentation, misclassifying background areas as potholes, whereas E-Net tends towards under-segmentation, failing to encapsulate entire pothole regions. The visual comparison robustly illustrates the divergence in model performance, with PoFormer’s segmented outputs demonstrating remarkable concordance with the ground truth. This fidelity suggests that PoFormer outstrips the comparative models in discerning the nuanced features of potholes, thereby substantiating its superior segmentation capabilities.

The superior performance of PoFormer over the benchmark models can be attributed to its hybrid architecture, which effectively combines the global feature-extraction capability of Transformers with the localized spatial precision of CNNs. Unlike CNN-only models, such as U-Net or E-Net, which mainly capture neighborhood-level textures, the Transformer encoder in PoFormer models long-range dependencies across the pavement surface, enabling it to differentiate potholes from other dark or irregular areas (e.g., shadows, patches). Meanwhile, the CNN-based decoder reconstructs sharp boundaries and fine surface details, which are often lost in Transformer-only architectures. This synergy results in more complete and accurate segmentation masks, as seen in Figure 9. Furthermore, PoFormer exhibits the lowest standard deviation in both mIoU and mPA, demonstrating greater stability and generalization across diverse environmental conditions. These findings confirm that integrating contextual reasoning with detailed spatial recovery is the key factor behind PoFormer’s improved segmentation performance.

## 4. Conclusions

In this work, a Transformer–CNN hybrid model, PoFormer, is proposed as an effective tool for segmentizing pavement potholes. It is an encoder–decoder structure where Transformer is used as the encoder and a CNN is utilized as the decoder. It can leverage the strengths of both network architectures, utilizing Transformer to capture long-range dependencies and contextual relationships in data and applying CNN as the decoder to extract spatial hierarchies of features. The model demonstrates a strong capability to overcome the limitations of traditional CNN-only methods, like limited contextual understanding and overfitting on textural features, and significantly improved the accuracy and robustness of pothole segmentation enabled by Transformer’s ability to capture complex dependencies and handle sequential data. We compared the PoFormer with other art-in-state models, including U-Net, E-Net, FCN, LRASPP, and AttuNet. This showed that PoFormer achieves superior performance metrics, with an mIoU of 77.23% and an mPA of 84.48%. These results substantiate the model’s capability to accurately segment potholes, which is essential for the development of intelligent transportation systems and infrastructure maintenance.

In addition, a heterogeneous dataset containing various resolutions of pothole images are collected in this work and posted on GitHub, which is designed to reflect a range of real-world conditions affecting pothole visibility and detection. This collection showcases various pothole scenarios, including different sizes, shapes, and severities, as well as a variety of environmental conditions such as wet and dry pavements. Most of the images contain more than one pothole, and each one can be a random size and irregular shape with wet or dry conditions, which demonstrates the strong heterogeneity features of pothole morphology distribution. This open source pothole image dataset can be used as a benchmark dataset to evaluate the upcoming models designed for pothole segmentation tasks. This is intended to contribute to the pavement defect segmentation and data science communities.

Although PoFormer demonstrates superior segmentation accuracy and robustness, several limitations should be noted. First, the Transformer encoder increases computational cost, which may restrict the model’s deployment on low-power or real-time inspection platforms. Second, under extremely low illumination, shadow interference, or heavy occlusion, performance may degrade due to insufficient visual cues. Third, the current framework focuses exclusively on pothole segmentation, and further modifications would be required to extend it to multi-type pavement distress detection. Despite these constraints, PoFormer is particularly suitable for offline pavement condition assessment, automated inspection reporting, and maintenance prioritization, where high segmentation accuracy and generalization are essential.

Future research directions include optimizing the model’s computational efficiency and extending its application to other pavement distress types such as cracks. The findings of this study provide a foundation for the advancement of automated pavement condition assessment techniques, with the potential to significantly impact the field of transportation infrastructure management. The contributions of this research extend beyond pothole segmentation. The integration of Transformers into pavement analysis tasks opens up new avenues for advanced computer vision techniques in transportation infrastructure management. The insights gained from this study can inform future developments in automated road condition assessment, maintenance planning, and resource optimization.

## Figures and Tables

**Table 1:** Comparative performance metrics of evaluated models.

Model	Precision	Recall	F_1_-Score
FCN	79.25	60.80	68.79
E-Net	83.77	66.87	74.31
LRASPP	86.08	71.31	78.0
U-Net	86.78	59.68	70.71
AttuNet	86.09	68.47	76.27
PoFormer	85.92	72.20	78.43

## Data Availability

The original data presented in the study are openly available in GitHub at https://github.com/tjboise/PoFormer (accessed on 2 November 2025).
